# Congenital Transmission of Chagas Disease in Latin American Immigrants in Switzerland

**DOI:** 10.3201/eid1504.080438

**Published:** 2009-04

**Authors:** Yves Jackson, Catherine Myers, Alessandro Diana, Hans-Peter Marti, Hans Wolff, François Chappuis, Louis Loutan, Alain Gervaix

**Affiliations:** Geneva University Hospitals and University of Geneva, Geneva, Switzerland (Y. Jackson, C. Myers, A. Diana, H. Wolff, F. Chappuis, L. Loutan, A. Gervaix); Swiss Tropical Institute, Basel, Switzerland (H.-P. Marti)

**Keywords:** Chagas disease, congenital, *Trypanosoma cruzi*, endemic, screening, Switzerland, pregnant women, Latin America, dispatch

## Abstract

International migration has changed the epidemiologic patterns of Chagas disease. Recently, 2 cases of Chagas disease transmitted from Latin American women to their newborns were diagnosed in Geneva, Switzerland. A retrospective study to detect Chagas disease showed a prevalence of 9.7% among 72 Latin American women tested during pregnancy in Switzerland.

Chagas disease, a zoonotic infection caused by *Trypanosoma cruzi*, is the most important endemic parasitic infection in Mexico and Central and South America because of the number of persons who become ill or die from this disease (*1*). An estimated 8–10 million persons are infected, and ≈14,000 persons die each year from Chagas disease (*1*,*2*). Historically, transmission by triatomine vectors has been the most common source of infection; however, the populations affected, transmission routes, and geographic distribution of Chagas disease cases have been greatly modified by urbanization and international migration. An estimated 14 million people from countries in which Chagas disease is endemic have moved to North America, Europe, Japan, and Australia. The number of persons currently infected by *T. cruzi* is probably >100,000 in the United States and >6,000 in Spain (*2*).

In Europe, vertical, transfusional, and transplantational routes have accounted for all cases of transmission. The risk for vertical transmission from an infected mother to her newborn is ≈5% (*3*). Vertical transmission is likely to go undetected in Europe because of lack of screening programs for at-risk pregnant women, who are usually in the long-lasting, chronic, asymptomatic phase of the disease and are unaware of their infection. An estimated 2,000 babies may have been born with *T. cruzi* infection in North America in recent years, and 2 cases of vertical transmission were recently reported from Spain (*4*–*6*). We report 2 additional cases of congenital infection with *T. cruzi*, detected in 2001 and 2006, at the Geneva University Hospitals in Switzerland. Subsequently, we conducted a retrospective serologic survey of pregnant Latin American immigrants to assess the potential for vertical transmission of Chagas disease in Switzerland.

## The Cases

**Figure 1:**
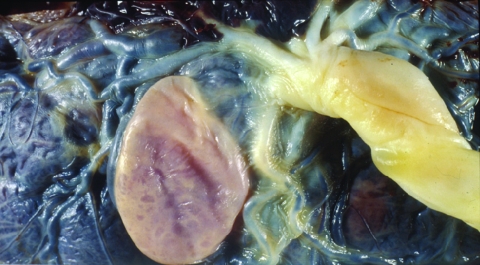
Fetal side of the placenta from Latin American pregnant woman who delivered her baby at Geneva University Hospitals, Geneva, Switzerland. A macroscopic subchorial liquid-filled cyst can be seen near the umbilical cord insertion.

In 2001, a 31-year-old woman from Santa Cruz, Bolivia, delivered a 2,860-g, full-term, apparently healthy baby at the Geneva University Hospitals after an uncomplicated pregnancy. Like most undocumented immigrants recently arrived in Switzerland, she had received no medical supervision during her pregnancy. She stated that a blood test for *T. cruzi*, conducted in Bolivia, had been negative. Macroscopic examination of the fetal side of the placenta showed a 3.5-cm, subchorial, liquid-filled cyst (Figure 1). Histopathologic examination showed disseminated chorioamnionitis and associated funiculitis with large numbers of nonflagellated parasites. A recent infection with *Toxoplasma gondii* was ruled out by serologic testing. Congenital *T. cruzi* infection was confirmed by a positive blood microscopic examination for the infant, a positive serologic test result for the mother (immunofluorescence assay using killed *T. cruzi* parasites, Swiss Tropical Institute, Basel, Switzerland), and a positive blood PCR with TCZ1/TCZ2 primers for both the mother and the newborn. Electrocardiogram and echocardiogram of the newborn showed no abnormalities. The newborn received nifurtimox (10 mg/kg/d for 60 days) without notable adverse effects. Parasitemia became undetectable at the end of treatment, and serologic test result at 1 year of age was negative. The mother refused to be treated, claiming that she was feeling fine.

In 2006, a 25-year-old woman arrived in Switzerland from Santa Cruz, Bolivia, when she was 5 months pregnant. She delivered a 2,480-g, premature but healthy baby at 34 weeks’ gestation at the Geneva University Hospitals. After discharge, histopathologic examination of the placenta showed funiculitis and chorioamnionitis with clusters of nonflagellated parasites. The mother had not been previously tested for *T. cruzi* but related that her father had died of Chagas disease–related heart complications. *T. cruzi* serologic testing by immunofluorescence was positive for the mother, and blood microscopic examination and PCR were positive for the newborn, confirming vertical transmission. Electrocardiogram and echocardiogram of the baby showed no abnormalities. The newborn began a 60-day course of nifurtimox (10 mg/kg/d) at 20 weeks of age and had no adverse effects. Blood PCR and serologic testing at 5 and 26 weeks after treatment was started, respectively, produced negative results. The mother was treated with nifurtimox after completion of breast-feeding and showed good tolerance to the drug.

## The Study

In response to these 2 cases, in 2007, a retrospective serologic survey for *T. cruzi* infection was performed on stored serum samples from 72 undocumented pregnant Latin American women who had received prenatal care at the Geneva University Hospitals during the previous year. Median age was 30 years (range 20–43), and countries of origin were Bolivia (n = 30), Brazil (n = 22), Peru (n = 6), Ecuador (n = 5), Colombia (n = 4), Chile (n = 2), Honduras (n = 1), and unknown (n = 2). Serum samples were tested by IFA using *T. cruzi* parasites from in vitro culture (Swiss Tropical Institute). No confirmatory test was available. Of the 72 samples, 7 (9.7%) were positive, most from Bolivian women (n = 5). The seroprevalence among Bolivian women was 16.6% (5/30), consistent with prevalence found by similar surveys conducted recently in Bolivian maternity hospitals (*7*). Limitations of the study include the small number of samples tested and lack of a confirmatory test as recommended by the World Health Organization.

## Conclusions

Only a small number of congenital cases of Chagas disease have been reported in countries in which this infection is nonendemic. The absence of routine screening programs for Chagas disease in pregnant women and newborns at risk most likely explains this low number, but other factors may be involved. Chagas disease affects immigrants, who frequently lack legal status and therefore experience difficulties (e.g., fear of deportation and financial and administrative constraints) in accessing quality healthcare during pregnancy. In Switzerland, undocumented immigrant women have poor access to medical supervision during pregnancy, so most consult a physician late in pregnancy or at time of delivery (*8*). Chagas disease is rare in Europe, and healthcare workers may simply not search for it, resulting in missed opportunities to diagnose the disease. In addition, up to two thirds of infected newborns are asymptomatic at birth, so congenital infection may go undetected if not actively sought.

Systematic screening of pregnant women at risk is likely to be beneficial in several ways. Treatment of infected mothers after completion of breast-feeding may reduce the risk for vertical transmission during subsequent pregnancies. Treatment of young women at the chronic, indeterminate stage of infection is likely to lower their risk for developing cardiac complications (*9*). Early screening and treatment of infected newborns are associated with high cure rates (*10*). Older children of mothers with newly diagnosed Chagas disease also benefit from screening and treatment (*11*). In addition, because immigrants with inadequate access to healthcare are at risk for being lost to follow-up after delivery, perinatal screening offers a good opportunity to screen other family members and offer treatment as needed.

Because most pregnant women receive their diagnosis during the chronic, asymptomatic stage of Chagas disease, screening with 2 sequential serologic tests is the most efficient strategy for detection of infection (*12*). PCR and parasitologic tests are ineffective for detection because they show lower sensitivity during this phase (*13*). In contrast, infected newborns usually have high levels of parasitemia. Therefore, microscopic techniques such as microhematocrit and concentration methods in umbilical cord blood have fairly high (>80%) sensitivity (*14*). PCR is more sensitive for detecting Chagas infection in infants than in adults; however, few laboratories perform *T. cruzi* PCR in Europe.

**Figure 2:**
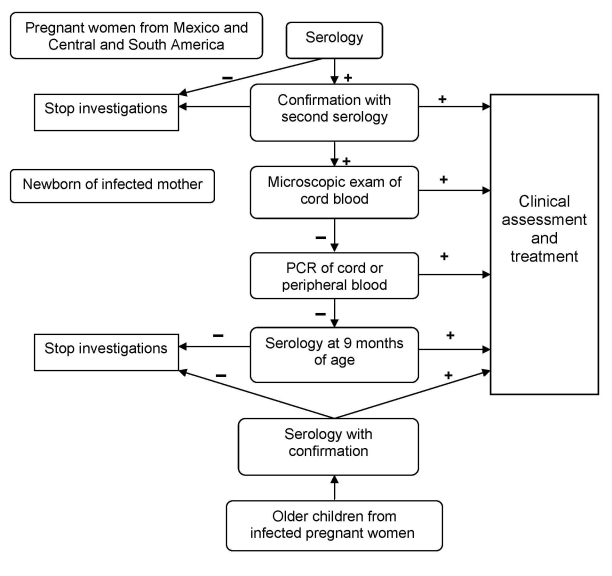
Algorithm for screening, diagnosis, and treatment of *Trypanosoma cruzi* congenital infection at Geneva University Hospitals, Geneva, Switzerland.

Launched in January 2008, a program of systematic Chagas disease screening of pregnant women at risk and of newborns delivered by infected mothers is under way at the Geneva University Hospitals (Figure 2). All pregnant women from Mexico and Central and South America are screened by serologic testing. Newborns of infected mothers are screened by microscopic examination of cord blood after concentration (microhematocrit, Strout's method) and, if negative, by PCR. If PCR is negative, serologic testing is performed when the child is 9 months of age. Blood cultures are not performed because of time needed to obtain results. Examination of the placenta, which is an unreliable screening method, is also not conducted for diagnostic purposes (*15*). All infected mothers (after completion of breast-feeding), newborns, and their siblings are offered treatment for this potentially fatal disease. Prenatal and delivery care of Latin American immigrants is an opportunity to screen for Chagas disease and its potential vertical transmission. This strategy will help address this emergent health problem in Europe.
